# Adenoid hypertrophy is directly associated with the severity of OSA in obese children: A pilot study

**DOI:** 10.1016/j.bjorl.2025.101694

**Published:** 2025-08-05

**Authors:** Victor Hugo da Costa Ferreira, Carolina Sponchiado Miura, Bruna de Alencar Custodio Lupoli, Denny Marcos Garcia, Bruno Carvalho Portes Lopes, Fernando Gustavo Stelzer, Fábio Lourenço Romano, Tabata Luna Garavazzo Tavares, Alan Luiz Eckeli, Fabiana Cardoso Pereira Valera

**Affiliations:** a Universidade de São Paulo (USP), Faculdade de Medicina de Ribeirão Preto (FMRP), Ribeirão Preto, SP, Brazil; b Universidade de São Paulo (USP), Faculdade de Medicina de Ribeirão Preto (FMRP), Departamento de Oftalmologia, Otorrinolaringologia e Cirurgia de Cabeça e Pescoço, Ribeirão Preto, SP, Brazil; c Universidade de São Paulo (USP), Faculdade de Medicina de Ribeirão Preto (FMRP), Departamento de Ortopedia e Anestesiologia, Ribeirão Preto, SP, Brazil; d Universidade de São Paulo (USP), Faculdade de Medicina de Ribeirão Preto (FMRP), Departamento de Neurologia e Ciências Comportamentais, Ribeirão Preto, SP, Brazil; e Universidade de São Paulo (USP), Faculdade de Odontologia de Ribeirão Preto (FORP), Departamento de Clínicas Pediátricas, Ortodontia, Ribeirão Preto, SP, Brazil

**Keywords:** Obstructive sleep apnea, Drug-induced sleep endoscopy, Children, Obesity

## Abstract

**Objective:**

This study aimed to evaluate which physical findings, observed during both awake and DISE examinations, correlate best with polysomnographic severity in obese children with Obstructive Sleep Apnea (OSA).

**Methods:**

Children diagnosed with pediatric OSA and obesity (BMI > 95th percentile) were evaluated. All underwent polysomnography to confirm the OSA diagnosis. Demographic data, physical exams, and ENT clinical evaluations were also collected. DISE (drug induced sleep endoscopy) was performed just before adenotonsillectomy, following Boudewyns' protocol to assess pharyngeal collapse.

**Results:**

Nineteen obese children with OSA were evaluated, with 37% showing adenoid hypertrophy (>75%) on nasal endoscopy, and 53% presented tonsillar hypertrophy on physical examination. The median AHI (apnea / hypopnea index) was 7 (range: 3; 17): 9 had mild, 2 moderate, and 8 severe OSA. Children with adenoid hypertrophy had higher arousal indices, AHI, REM (rapid eye movement) AHI, OAHI (obstructive AHI) and lower minimum saturation compared to those without obstruction, in both awake and DISE evaluations. The presence of other factors, such as tonsillar hypertrophy, tongue base obstruction, palatal and epiglottic collapses, or hypotonia, was not associated with OSA severity or polysomnographic findings in either awake or DISE exams.

**Conclusion:**

In our cohort, adenoid hypertrophy was the factor most strongly correlated with OSA severity in obese children. This correlation was consistent between awake physical examination and DISE findings.

**Level of evidence:**

3.

## Highlights

• Adenoid hypertrophy is intrinsically related to OSA severity in obese children.
• DISE should be ideally recommended for children without adenotonsillar hypertrophy.
• The parameters in awake and DISE evaluations were equally related to OSA severity.

## Introduction

Obstructive Sleep Apnea (OSA) is characterized by a reduction or complete cessation of breathing, associated with increased respiratory effort during sleep.^1^^,^^2^ The prevalence of this disease in childhood is estimated to be between 1% and 3%. Pediatric OSA has a significant impact, as it is linked to an increased risk of growth deficits, enuresis, poor academic performance, and behavioral disorders. Additionally, there is a correlation between OSA and the frequency of attention/hyperactivity disorders, so much so that many patients initially diagnosed with Attention Deficit Hyperactivity Disorder (ADHD) are, in fact, untreated OSA cases.^3^

Several key factors are associated with childhood OSA, such as adenotonsillar hypertrophy, craniofacial abnormalities, obesity, and pharyngeal neuromotor dysfunction.^4^ Among these, obesity plays an additional role, as it is also the main factor linked to the persistence of OSA after adenotonsillectomy, known as residual OSA.^5^ While it is well established that obesity in children is associated with a higher frequency and severity of OSA, the predominant obstruction sites in obese children have been underexplored in the literature.

The gold standard exam for diagnosing OSA is overnight polysomnography, which can identify respiratory events and quantify the severity of OSA. Once the diagnosis is confirmed and the severity of OSA is staged, it is essential to identify the associated factors and the anatomical sites most prone to obstruction. This allows for the development of a more targeted treatment strategy.

The assessment of the upper airway through nasoendoscopy while the child is awake does not reflect the muscle tone present during sleep. In this regard, endoscopic visualization of the pharynx under sedation, known as Drug-Induced Sleep Endoscopy (DISE), provides new opportunities to better evaluate the factors contributing to OSA. DISE specifically identifies the sites of obstruction in the upper airway during light sedation, which mimics sleep, guiding a more focused treatment approach for OSA patients. In children, DISE is mainly indicated for those with associated diseases, high risk of residual OSA, children without clear signs of obstruction during physical examination and in cases of residual OSA after adenotonsillectomy.^1^^,^^6^^,^^7^ However, there is no consensus regarding its use for routine pre-surgical evaluations.

Therefore, assessing the relationship between DISE findings and OSA severity is crucial for more precise and evidence-based indications for surgical treatment. The present study aims to evaluate which sites are most affected in obese children immediately before adenotonsillectomy and how these sites correlate with the severity of OSA.

## Methods

### Patients

This study evaluated children under follow-up at the Mouth Breather Center (CERB) of the Clinics Hospital, Medical School of Ribeirão Preto - University of São Paulo (HCFMRP-USP) between October 2021 and March 2024. The study included patients of both genders who were being followed at CERB for pediatric OSA and obesity (BMI – Body Mass Index >95th percentile for age, according to WHO standards) and had indications for adenotonsillectomy.

### Sleep study

As an inclusion criterion, all children underwent a clinical evaluation followed by overnight polysomnography at the Sleep Laboratory of the Hospital (HCFMRP-USP). The criteria from the American Academy of Sleep Medicine (AASM) were applied, considering Obstructive Apnea-Hypopnea Index (OAHI) greater than 1 associated with clinical symptoms to diagnose pediatric OSA.^8^ For severity classification, cases with OAHI between 1 and 4.9 were considered mild OSA; those with OAHI between 5 to 9.9 were considered moderate, and severe OSA was considered when OAHI was greater than 10. Only children with OAHI greater than 1 were invited to participate in the study.

Polysomnography was performed using a digital polygraph (Neurovirtual, Brazil). Data included the use of an electroencephalogram (F3-M1, F4-M2, C3-M1, C4-M1, O1-M1, O2-M2), bilateral electrooculogram, electrocardiogram (modified V2 lead), surface electromyography of the chin and submental muscles, surface electrodes on both anterior tibialis muscles, and digital video recorded with an infrared camera synchronized with the polysomnography data. Respiration was monitored as follows: airflow was measured with a nasal pressure transducer and nasal thermistor airflow sensor; thoracic and abdominal effort was measured using inductive plethysmographic belts; arterial oxygen saturation was measured with pulse oximetry; snoring sounds were monitored with a snore microphone, and body position was tracked using a position sensor. All technical procedures followed the “AASM Manual for the Scoring of Sleep and Associated Events: Rules, Terminology, and Technical Specifications v2.0”.^9^ According to the Manual, hypopnea is defined as a reduction in airflow of 50% or more, lasting for at least two missing breaths, and associated with either a decrease in O_2_ saturation of 3% or more, or an arousal.

The following exclusion criteria were applied: Syndromic patients, those with craniofacial abnormalities or those neurological developmental delay; Patients with severe asthma.

### Awake examination

After confirming all inclusion and exclusion criteria and obtaining the parents' or guardians' signed consent and assent forms, the following evaluations were performed:

• Pediatric Sleep Questionnaire (PSQ): A Brazilian Portuguese-validated version of the Pediatric Sleep Questionnaire.^10^
• Clinical ENT assessment: This included oroscopy, with classification according to the Brodsky scale (for tonsil size evaluation) and Mallampati scale.
• Outpatient nasal endoscopy was conducted while the patient was awake to assess the percentage of nasopharyngeal obstruction caused by the adenoids.
• Orthodontic evaluation, made by an Orthodontics Professor.

### Drug induced sleep endoscopy

As part of the study protocol, on the day of surgery, the children underwent DISE (Drug-Induced Sleep Endoscopy) immediately before the surgery. Both the DISE and the adenotonsillectomy were performed in the surgical center of the Hospital (HCFMRP), under the supervision of the same anesthesiologist and otorhinolaryngologists.

DISE was performed during the induction of anesthesia without premedication. Sedation was initiated by the anesthesiologist using propofol for both induction (1–2 mg/kg bolus) and maintenance (0.2 mg/kg/min), with the sedation level monitored via the Bispectral Index (BIS). An adequate anesthetic depth was considered when BIS values were between 50 and 70. At this point, two puffs of 10% lidocaine spray were applied to each nostril to minimize discomfort caused by the nasoendoscope passing through the nasal structures. Various areas of the nasal passages and pharynx were evaluated following the Boudewyns protocol.^11^ The observed findings included pharyngeal collapse sites, structures involved in the collapse ‒ such as the adenoids, tonsils, base of the tongue, palate, epiglottis, hypotonia, and laryngomalacia ‒ and the collapse pattern (anteroposterior, lateral, or concentric).

### Data collection

All collected data were recorded in a spreadsheet on the institution's RedCap system.^12^ For the current research, the following data were selected:

• Demographic data: gender, age, orthodontic abnormalities (such as high-arched palate, open bite, and crossbite), and the anteroposterior classification of the upper and lower first molars.
• Physical examination data: weight, height, and BMI, according to age, based on WHO guidelines.
• PSQ (Pediatric Sleep Questionnaire): A questionnaire assessing the quality of life in children with sleep disorders, translated for use in Brazil.
• ENT examination data: conducted in the clinic, including tonsil size according to the Brodsky scale and adenoid size on nasal endoscopy.
• Polysomnography: with special emphasis on sleep efficiency, sleep architecture, arousal index, and OAHI.
• DISE (Drug-Induced Sleep Endoscopy): Based on the Boudewyns scale.^11^

### Statistical analysis

The data were presented as medians with Interquartile Ranges (IQR) for numerical variables, and as frequencies and percentages for categorical variables. Comparisons between subgroups were conducted using the Chi-square test and Mann-Whitney test. All analyses were performed using R software (R Core Team, 2024), with a significance level set at 5%.

### Ethics

This project was approved by local IRB, under the number CAAE 84499418.9.0000.5440.

## Results

Nineteen patients aged 4–13 years (median age 8.0 [6.0; 10.0]), underwent DISE on the same day as their adenotonsillectomy. The median BMI of the patients was 28.8 kg/m^2^ (26.5; 32.2). Seventeen of the nineteen children (89%) had a Z-score of +3, and two had a Z-score of +2. Fourteen of the children (74%) were male.

The primary indication for adenotonsillectomy was loud and persistent snoring accompanied by mouth breathing, which was present in all patients. All participants had OSA confirmed by PSG, as detailed below. Additionally, seven patients exhibited enuresis, while daytime somnolence was reported in 10 children. Fourteen children were described as easily distracted and unable to complete their activities by their parents. Five patients also had a history of recurrent tonsillitis and one child presented with recurrent rhinosinusitis.

Among the participants, 53% presented with obstructive tonsils upon physical examination (Brodsky Grade III or IV), and 37% had adenoid obstruction (defined as greater than 75% obstruction of the nasopharynx on nasal endoscopy). Orthodontic abnormalities were present in 8 of the 19 patients, with a high-arched palate being the most common, found in 7 patients. One child (aged 4-years) had Class II occlusion (Angle classification), while two children (both aged 10 years) had Class III occlusion.

The median PSQ score was 14.0 (10.5; 17.0). A PSQ score greater than 9 is considered indicative of pediatric OSA. The demographic data are further detailed in Table 1.

**Table 1:** Descriptive data of the studied sample.

Variable	n = 19^a^
Age	8.00 (6.00; 10.00)
Gender	
F	5 (26%)
M	14 (74%)
Tonsils	
Non-obstructive	9 (47%)
Obstructive	10 (53%)
Adenoid size	
Non-obstructive	12 (63%)
75%+	7 (37%)
Orthodontic abnormalities	
None	11 (58%)
Open bite	1 (5.3%)
High-arched palate * 1 patient with crossbite	7 (36.8%)
Orthodontic abnormalities	
No	11 (58%)
Yes	8 (42%)
Angle’s classification	
1	15 (79%)
2	1 (5.3%)
3	3 (16%)
BMI	28.8 (26.5; 32.2)
Z-score	
2+	2 (11%)
3+	17 (89%)
Total PSQ	14.0 (10.5; 17.0)

a Median (IQR); n (%).

Among the data obtained from polysomnography, the median sleep efficiency was 87% (81; 92), with a median arousal index of 17 (11; 21). The median average oxygen saturation was 96% (95.50; 97.05), while the median minimum oxygen saturation was 88% (81.5; 91.5). The median OAHI was 7 (3; 17), with 9 patients classified as having mild OSA, 2 with moderate OSA, and 8 with severe OSA (Table 2).

**Table 2:** Descriptive data of polysomnography.

Variable	n = 19^a^
Sleep efficiency	87 (81; 92)
Arousal index	17 (11; 21)
%N1	5.6 (4.1; 9.8)
%N2	47 (41; 51)
%N3	30 (25; 38)
%REM	15.6 (11.9; 18.2)
OAHI	7 (3; 17)
AHI	8 (3; 17)
REM AHI	7 (3; 31)
Minimum saturation	96.00 (95.50; 97.05)
Average saturation	88.0 (81.5; 91.5)

a Median (IQR); n (%).

**Fig. 1:**
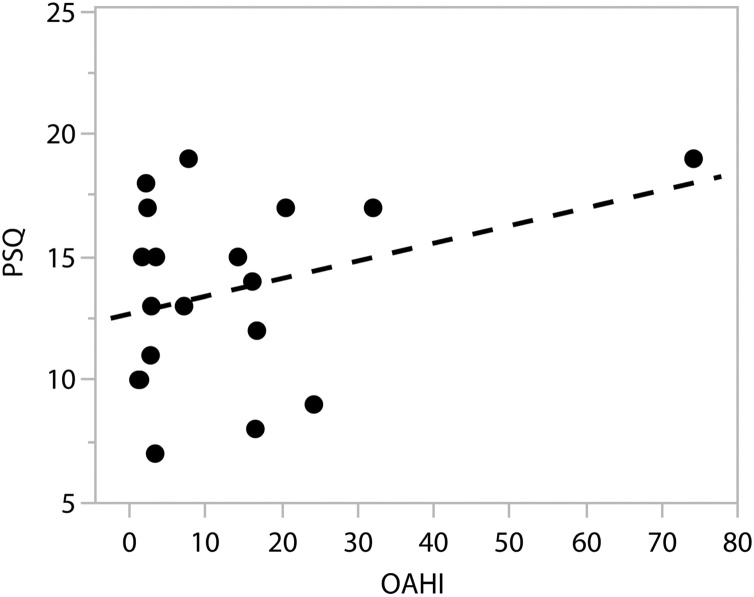
Association between total PSQ and OAHI. A correlation of 0.33 is observed (95% CI -0.14‒0.68, p not significant).

The PSQ was a significant predictor of OSA in children, but it lacked the ability to distinguish between the severity levels of OSA, as no direct correlation was found between the OAHI and the total PSQ score (Fig. 1).

In terms of DISE findings, adenoid obstruction (> 75%) was identified in 32% of patients, while tonsillar obstruction was present in 68%. Tongue base obstruction was observed in 32% of cases, palatal collapse in 47%, and epiglottic collapse in 37%. Hypotonia was detected in 32% of patients, with laryngomalacia occurring in only one child (Table 3).

**Table 3:** Descriptive data of DISE.

Variables	n = 19^a^; n (%)
Adenoid	
0‒75	13 (68%)
75+	6 (32%)
Tonsils	
Non-obstructive	6 (32%)
Obstructive	13 (68%)
Base of the tongue	
Non-obstructive	13 (68%)
Obstructive	6 (32%)
Palate	
Absence of collapse	10 (53%)
Presence of collapse	9 (47%)
Epiglote	
Absence of collapse	12 (63%)
Presence of collapse	7 (37%)
Hypotonia	
Absence	13 (68%)
Presence	6 (32%)
Laryngomalacia	
Absence	18 (95%)
Presence	1 (5.3%)

a Median (IQR; interquartile range).

We initially observed which factors in the awake physical examination were most associated with the severity of pediatric OSA. No association was found between occlusal alterations or tonsillar obstruction and the severity of any polysomnographic parameters evaluated.

When analyzing the relationship between adenoid obstruction ‒ identified in the physical examination ‒ and the polysomnography data, we observed that patients with adenoid obstruction showed significantly higher arousal index [21 (18; 31) vs. 16 (6; 18); p < 0.05], OAHI [21 (11; 28) vs. 3 (2; 7); p < 0.005], AHI [21 (11; 29) vs. 3 (2; 7); p < 0.005], and REM AHI [37 (28; 47) vs. 4 (2; 6); p < 0.005], in addition to lower minimum saturation [85.0 (79.5; 87.5) vs. 90.5 (85.8; 92.3); p < 0.05] compared to the group without adenoid obstruction. There was no significant difference in the PSQ scores between children with and without adenoid hypertrophy (Table 4).

**Table 4:** Data related to PSQ and polysomnography according to adenoid size in physical examination.

	Adenoid size		p-valor^b^
	0‒75 (n = 12)^a^	75+ (n = 7)^a^	
Total PSQ	12.5 (10.0; 15.0)	17.0 (14.0; 18.0)	0.089
Sleep efficiency	87 (84; 91)	82 (79; 91)	0.5
Arousal index	16 (6; 18)	21 (18; 31)	0.047
%N1	6.2 (3.7; 8.2)	5.6 (4.5; 9.8)	>0.9
%N2	46 (40; 51)	47 (43; 56)	0.6
%N3	30 (25; 37)	30 (28; 35)	0.9
%REM	16.4 (12.7; 19.3)	13.5 (10.1; 15.7)	0.2
OAHI	3 (2; 7)	21 (11; 28)	0.004
AHI	3 (2; 7)	21 (11; 29)	0.004
REM AHI	4 (2; 6)	37 (28; 47)	0.004
Average saturation	96.60 (96.00; 97.20)	96.00 (95.00; 96.50)	0.2
Minimum saturation	90.5 (85.8; 92.3)	85.0 (79.5, 87.5)	0.038

a Median (IQR, Interquartile Range).

b Mann–Whitney *U* Test.

We then evaluated whether DISE could provide additional data related to the predominant obstructive site in relation to OSA severity. For this analysis, we considered only adenoid, tonsillar, and tongue base obstruction, as well as the presence or absence of hypotonia, as the other parameters were present in a very small proportion of children. No significant differences were observed between the groups with and without tonsillar and tongue base obstruction, nor between those with and without associated hypotonia (data not shown).

However, when analyzing the relationship between adenoid obstruction ‒ verified through DISE ‒ and polysomnography data, we observed that patients with adenoid obstruction had significantly higher OAHI [22 (11; 30) vs. 3 (2; 14); p < 0.01], AHI [22 (11; 31) vs. 3 (2; 15); p < 0.01], and REM AHI [32 (27; 49) vs. 5 (2; 9); p < 0.05] compared to the group without adenoid obstruction. Again, no significant difference was found in the PSQ scores between children with and without adenoid hypertrophy (Table 5).

**Table 5:** Data related to PSQ and polysomnography according to adenoid size in DISE.

Variable	Adenoid size in DISE		p-value^b^
	0‒75 (n = 13)^a^	75+ (n = 6)^a^	
Total PSQ	13.0 (10.0; 15.0)	17.0 (14.0; 18.5)	0.11
Sleep efficiency	87 (84; 93)	81 (79; 87)	0.12
Arousal index	16 (6; 19)	20 (18; 35)	0.10
%N1	5.6 (3.2; 7.4)	7.7 (4.9; 9.9)	0.5
%N2	49 (40; 51)	45 (42; 50)	>0.9
%N3	30 (25; 37)	30 (27; 37)	0.9
%REM	15.6 (10.7; 18.3)	14.6 (13.2; 15.8)	0.6
OAHI	3 (2; 14)	22 (11; 30)	0.005
AHI	3 (2; 15)	22 (11; 31)	0.005
REM AHI	5 (2; 9)	32 (27; 49)	0.014
Average saturation	96.30 (96.00; 97.10)	95.50 (95.00; 96.75)	0.2
Minimum saturation	90.0 (82.0; 92.0)	86.0 (79.8; 87.8)	0.094

a Median (IQR, Interquartile Range).

b Mann–Whitney *U* Test.

## Discussion

Obstructive Sleep Apnea (OSA) in obese children can affect up to 60% of this population,^13^ a prevalence significantly higher than the 1%–3% reported for the entire pediatric population. The increased incidence of OSA in obese children, along with the severity of the condition, is associated with various factors, including a higher prevalence of adenotonsillar obstruction linked to local and systemic inflammation, increased central adiposity, reduced residual lung capacity, and alterations in respiratory drive. These factors may explain the differences observed between obese and non-obese children.^14^

In our study, the median Pediatric Sleep Questionnaire (PSQ) score was 14 (10.5; 17), which exceeds the cutoff value of 9, known for its high sensitivity and specificity in diagnosing OSA in children.^10^ Furthermore, no significant relationship was found between the total PSQ score and the severity of OSA observed in polysomnography, suggesting that the PSQ serves primarily as a diagnostic screening tool rather than a predictor of severity.

In this study, while patients were awake, we observed a prevalence of tonsillar obstruction (53%) compared to adenoid obstruction (37%). Our findings align with existing literature; Hasuneh et al. reported a strong association between tonsillar hypertrophy and the presence of OSA, while the frequency of adenoid hypertrophy was lower (RR = 3.55; 95% CI = 1.77–7.11 for tonsillar vs. RR = 1.63; 95% CI 1.09–2.42 for adenoid; p < 0.01).^15^ However, we noted that the severity of OSA was more directly related to adenoid hypertrophy than to tonsillar obstruction, which is consistent with findings reported by Tagaya et al.^16^ Thus, while tonsillar hypertrophy may have a higher frequency association with OSA, adenoid hypertrophy appears to be more closely related to its severity in the findings observed in awake obese children.

Considering that obese children may experience narrowing of the pharynx due to fat deposits in the pharyngeal structures or neuromuscular hypotonia,^17^ we hypothesize that the pattern of pharyngeal collapse in this group could differ between patients who are awake and those undergoing Drug-Induced Sleep Endoscopy (DISE). DISE is primarily used to evaluate the degree, site, and pattern of obstruction related to Obstructive Sleep Apnea (OSA) and helps identify mechanisms of pharyngeal collapse during light sedation-induced sleep. Studies have demonstrated its effectiveness as an adjunct in the surgical planning of adult patients with OSA, enabling a more individualized approach.^18^^,^^19^

In pediatric patients, DISE is particularly indicated for those with residual OSA after adenotonsillectomy,^17^ or in children with associated diseases that could increase the chance of residual OSA. Its role in preoperative evaluation remains elusive. Given that obese children are at a higher risk for severe OSA and for the persistence of OSA following adenotonsillectomy, we hypothesized that these patients would be more likely to exhibit hypotonia during sleep, suggesting that the pattern of pharyngeal collapse could differ during DISE. However, while 32% of patients demonstrated hypotonia of the pharyngeal muscles during DISE, this factor did not influence the severity of OSA in the current study.

Similar to the findings in awake patients, the only factor that showed a correlation with the severity of Obstructive Sleep Apnea (OSA) during Drug-Induced Sleep Endoscopy (DISE) was adenoid obstruction. This reinforces the idea that the adenoids may be a significant determinant of OSA compared to the other sites investigated.

There were no statistically significant differences in polysomnographic findings between patients with and without obstruction of the tongue base or tonsils during DISE. Additionally, the presence of collapse in the epiglottis or palate did not serve as predictors of OSA severity compared to patients without such findings. Hypotonia was also not associated with alterations in polysomnographic parameters in this study.

Interestingly, there was no significant difference between the assessment of adenoid obstruction via physical examination and DISE. We believe this finding may be attributed to the fact that this parameter does not significantly change between the two evaluations. Given the results, it seems that DISE may not play a role in investigating OSA in children with adenotonsillar hypertrophy, at least not in predicting the severity of OSA. This finding reinforces the current primary indications for DISE in children, specifically for those with persistent OSA following adenotonsillectomy or in children without evident obstructive factors despite the presence of OSA.^1^^,^^6^^,^^7^ This result stresses that the main purpose of DISE is to evaluate neuromuscular factors associated with OSA rather than obstructive factors.

It is important to note that the sample size in this study is relatively small, and further research may be necessary to confirm these findings. Additionally, while DISE may not have been essential in establishing a correlation with OSA severity, there is still the possibility of an association between DISE findings and the persistence of OSA following surgery, which will be evaluated in future studies.

Nevertheless, the results obtained reinforce the primary indications for DISE in the pediatric population. It should preferably be indicated for children after adenotonsillectomy or for those with OSA who do not exhibit any obvious obstructive factors. This latter indication has garnered unanimous consensus among specialists.

## Conclusion

In our cohort, adenoid hypertrophy was the finding most strongly correlated with the severity of OSA in obese children. Due to this finding, we did not observe a significant difference in this relationship during the physical examination, whether in the assessment of the awake patient or during DISE.

## Funding

FAPESP (grant 2023/13261-9 to V.H.C.F) and CNPq (process 405265/2018-2 and grant PQ1D 302384/2022-7 to F C.P.V.).

## Declaration of competing interest

All the authors declare they don’t have any additional financial or personal conflict of interest directly related to the article, apart from the institutions that funded this project.
